# Deep Learning‐Based Skin Lesion Classification: A CNN Approach on High‐Frequency Ultrasound Imaging

**DOI:** 10.1002/jum.70125

**Published:** 2025-11-20

**Authors:** Isabela Rocha Veiga da Silva, André Gonçalves Jardim, Giulia Rita de Souza Faés, Thatiane Alves Pianoschi Alva, Carla Diniz Lopes Becker, Viviane Rodrigues Botelho

**Affiliations:** ^1^ Federal University of Health Sciences of Porto Alegre Porto Alegre Brazil

**Keywords:** convolutional neural networks, HFUS, skin neoplasms, ultrasonography

## Abstract

High‐frequency ultrasound (HFUS) is valuable for assessing skin lesions, supporting diagnosis, treatment monitoring, and surgical planning. This study evaluates deep learning models for binary classification of HFUS images acquired in B‐mode and Doppler mode. Two single‐input CNNs were trained with each modality, while Unity and Cascade architectures combined both. The HFUS‐Doppler model achieved the best performance (95.0% accuracy, AUC 0.98), followed by Unity (90.5% accuracy, AUC 0.97). Cascade showed lower accuracy but greater confidence in malignant predictions. Probability distribution analysis revealed differences in model certainty near the decision threshold. Results indicate that combining B‐mode and Doppler can enhance diagnostic performance, depending on network design and data quality, supporting the potential of customized deep learning for non‐invasive HFUS‐based skin lesion classification.

**Abbreviations**
AI: Artificial Intelligence
AUC: area under the curve
CNNs: convolutional neural networks
HFUS: high‐frequency ultrasound
MLP: Multilayer Perceptron
ROC: receiver operating characteristic
XGBoost: Extreme Gradient Boosting

## Introduction

Over the past few decades, ultrasound has played an essential role in dermatological applications, with technological advances allowing greater detail of skin structures. High‐frequency ultrasound (HFUS), characterized by the use of frequencies above 15 MHz, has become a valuable tool in the diagnosis and management of skin disease. This technique provides high‐resolution images from the epidermis to the deep fascia and is widely used to assess skin thickness, neoplasia, inflammatory disease and for cosmetic purposes.^1^, ^2^, ^3^

The development of advanced equipment operating at frequencies up to 50 MHz has enabled detailed differentiation between skin layers and analysis of superficial structures such as skin appendages and blood vessels.^2^ These advances have made it possible to use HFUS to characterize dermatological diseases, monitor the effectiveness of treatments and plan surgical interventions.^2^, ^3^, ^4^ In addition, the integration of color Doppler with HFUS allows real‐time analysis of blood flow and vessel morphology, extending its applicability to dermatological conditions associated with vascular changes. This combination has proved particularly useful in differentiating benign from malignant lesions and for assessing the neoplasms' aggressiveness.^1^, ^2^, ^3^

The increase in the use of Artificial Intelligence (AI) as a diagnostic support tool is striking, particularly the rise in the use of convolutional neural networks (CNNs) to detect patterns and abnormalities in medical images, including ultrasound. With the advancement of AI, deep learning algorithms have shown promising performance in dermatological ultrasound analysis.^4^, ^5^, ^6^, ^7^, ^8^ Studies suggest that these tools are able to classify benign and malignant lesions with accuracy comparable to experienced dermatologists, even when trained on limited databases.^2^, ^4^

Recent studies on the application of neural networks to dermatological ultrasound include Laverde‐Saad et al's^4^ work, which represents a promising step toward the integration of AI in clinical practice. The EfficientNet B4 model developed by the authors achieved an accuracy of 77.1% in distinguishing between benign and malignant lesions, a relevant result that highlights the potential of deep learning in this field. The inclusion of additional performance metrics, such as learning curves, confusion matrix, and ROC curves, would contribute to a more comprehensive understanding of the model's predictive capacity. Furthermore, future studies could benefit from comparing different neural network architectures to ensure the most effective approach is being used. Such methodological enhancements are important to strengthen the robustness and clinical applicability of AI‐based solutions in dermatology.

In this context, this study aims to explore and evaluate computer vision methods applied to the classification of dermatological ultrasound images. The objective is to investigate how these technologies can complement and improve diagnostic practice, thus contributing to the advancement of image‐based dermatology by developing different architectures of convolutional neural networks for binary classification using the same HFUS image database introduced by Laverde‐Saad et al.^4^

## Materials and Methods

This section describes the following: 1) the dataset used; 2) the organization of the dataset; 3) the CNN architectures built; and 4) the metrics used to evaluate the architectures.

### Dataset

The dataset used in this study was originally described by Laverde‐Saad et al^4^ and made publicly available on the *Kaggle* platform.^9^ Formal authorization to use the dataset in this research was obtained from the original authors. This dataset consists of 202 data instances relating to skin lesions assessed by HFUS in both B‐mode and Doppler modalities. As described by Laverde‐Saad et al,^4^ the images were acquired according to the DERMUS protocol.^10^

- *A B‐mode image*: Grey scale image showing the morphological pattern of the lesion;
- *A color Doppler image*: Image highlighting the vascularization associated with the lesion;
- *Label column*: Binary classification of the lesion as benign or malignant;
- *Dx Column*: Indicates the specific classification of the tumor type according to the clinical categorization;
- *Freq column*: Contains information on the frequency of the transducer used to acquire the images. As described by Laverde‐Saad et al,^4^ the images were acquired with 10–22 MHz and 18 MHz transducers, reflecting the different levels of resolution and penetration applied during data acquisition.

**Figure 1:**
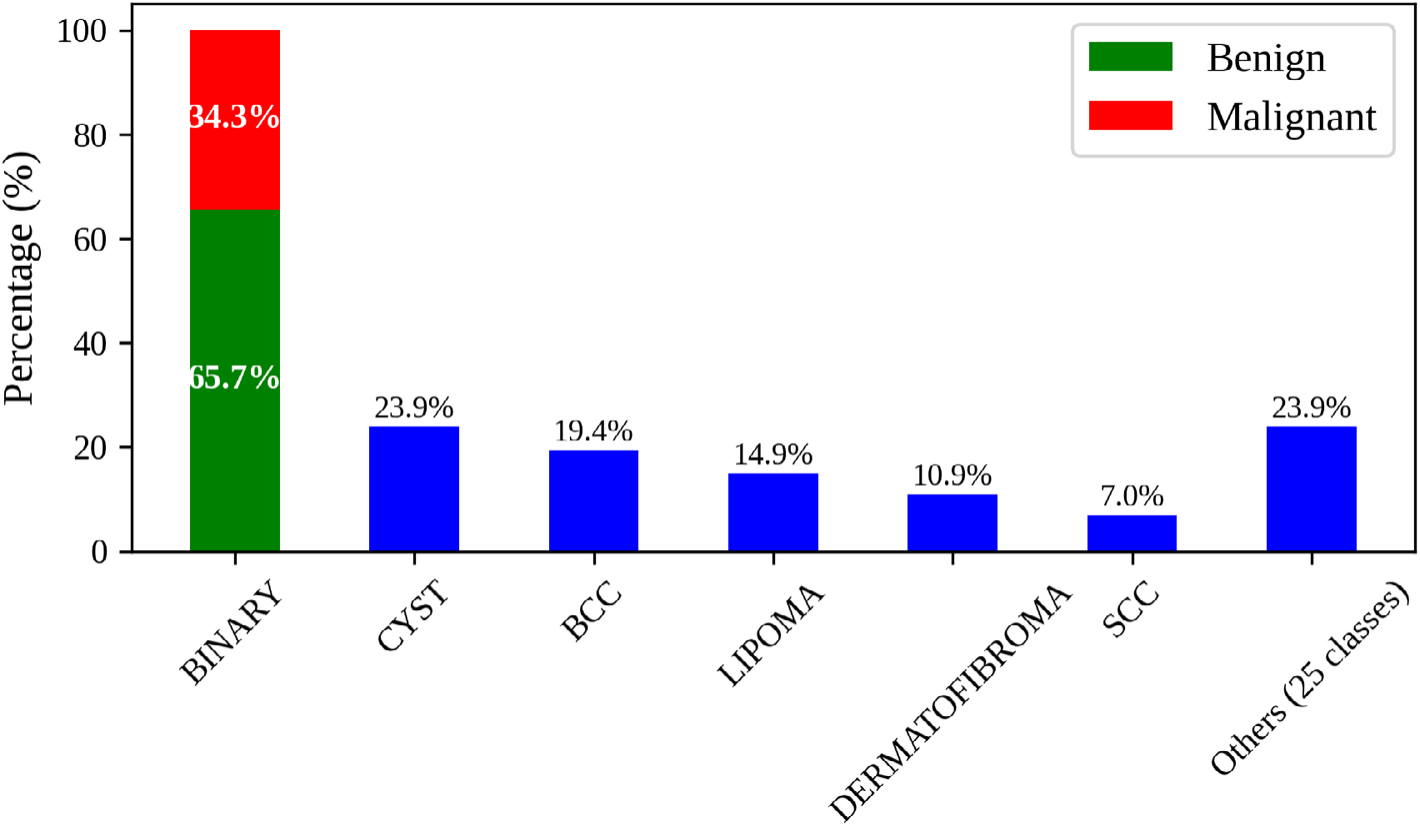
Distribution of classes in the HFUS image dataset of skin lesions. The left bar shows the binary distribution of lesions categorized as either benign (65.7%) or malignant (34.3%). The blue bars show the multi‐class distribution, including cysts (23.9%), basal cell carcinomas (BCCs) (19.4%), lipomas (14.9%), dermatofibromas (10.9%), squamous cell carcinomas (SCCs) (7.0%), and other lesion types (23.9%).

As shown in Figure 1, the dataset is significantly imbalanced, with a few classes containing most of the samples while others are underrepresented. This distribution limits the training and generalization capacity of deep learning models, making the dataset unsuitable for robust multi‐class classification under the current conditions.

### Dataset Organization

The dataset was carefully organized in multiple stages to ensure the consistency and quality of the information and images used in the models. First, three specific changes were made to the data table:

1. The names of the images at positions 57, 59, and 90 of the table have been corrected due to the inversion between acquisition modes, with B‐mode listed as Doppler and vice versa;
2. Adjustments have also been made in the formatting of the file names to ensure consistency, with the changes applied to the position images in Tables 31, 32, 106, 107, 110, and 137;
3. The instances at positions 6, 62, 74, and 121 were excluded because both images were acquired in Doppler mode, which does not meet the criteria of the dataset.

**Figure 2:**
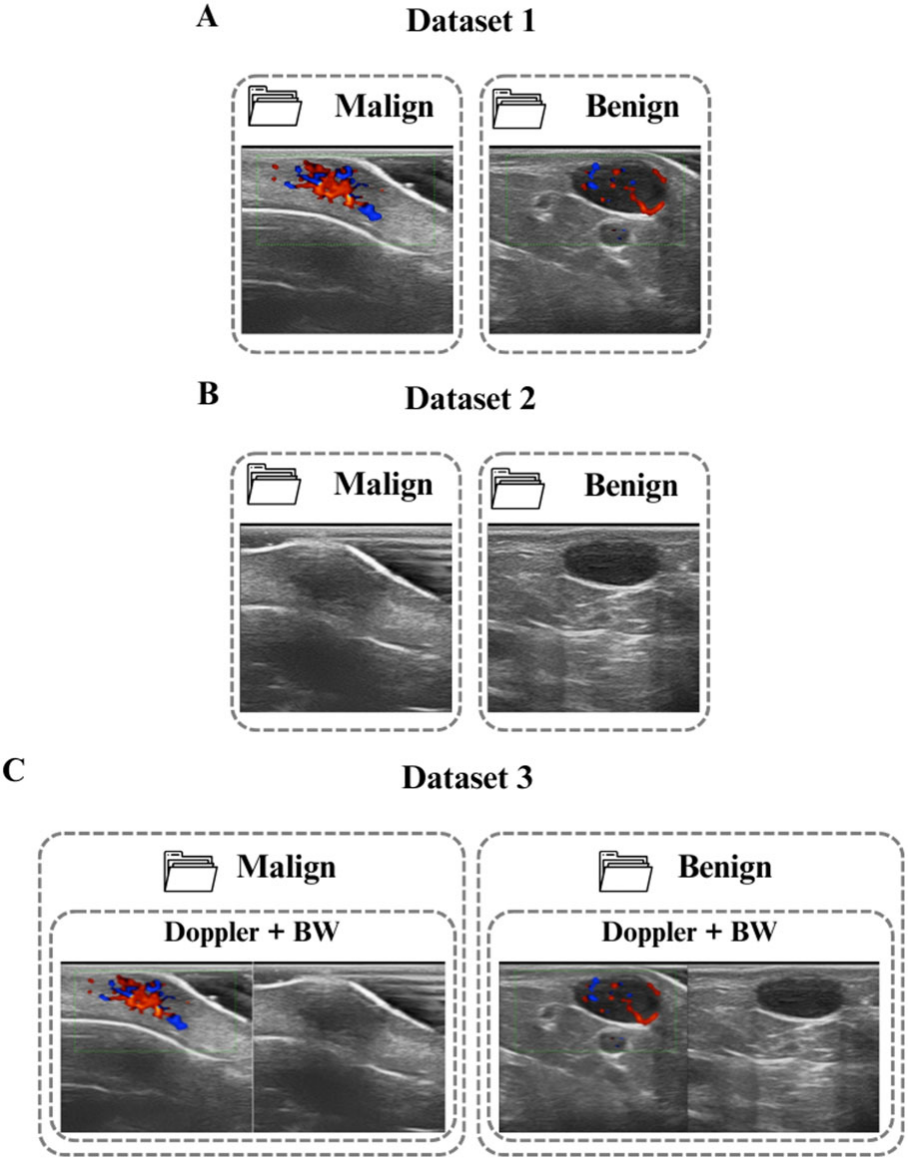
Organization of the datasets used to train the proposed architectures. **A**, Dataset 1 includes 224 × 224 pixel images in Doppler mode, classified as malignant or benign. **B**, Dataset 2 comprises B‐mode images, which are also classified as malignant or benign and have a size of 224 × 224 pixels. **C**, Dataset 3 comprises images created by combining Doppler and B modes. These images have dimensions of 224 × 448 pixels and maintain the same binary categorization as the previous datasets.

All images were resized to 224 × 224 pixels with three channels (RGB) to ensure compatibility with the convolutional network architectures used. Following the dataset organization stage, the images from the original dataset were organized into three different datasets according to the architecture, as shown in Figure 2. Dataset 1 consists of Doppler mode images only, which are separated according to their classification label. Dataset 2 is similar to the first; the difference is that it contains only B‐mode images. Dataset 3 combines both B‐mode and Doppler images by placing them side by side (horizontally), resulting in a new image with a final resolution of 224 × 448 pixels, maintaining three RGB channels.

### CNNs Architecture

This study applies four neural network‐based strategies to classify dermatological ultrasound images. To train the proposed architectures, the total amount of data available in each dataset was divided into three subsets: training, validation and testing sets. Initially, 10% of the data was set aside to form the test set which is used exclusively to evaluate the models' final performance, after full training. This set provides an independent metric for checking the model's ability to generalize to new data. The remaining data was subdivided into a training set, where the models adjust their parameters by learning from the patterns in the images, and a validation set, which monitors performance during training and helps to avoid problems such as overfitting. This balanced split ensures a fair and consistent assessment of the models whereas preserving the integrity of the test set for final analysis.

#### HFUS‐Doppler and ‐BW Architectures

The architectures used for Datasets 1 and 2 were based on simple convolutional networks that were designed to classify benign and malignant lesions as binary. Although the models were trained separately, the two networks have the same structure and differ only in their input data: Doppler mode images for HFUS‐Doppler and B‐mode images for HFUS‐BW. The first strategy, HFUS‐Doppler, uses Dataset 1 (Figure 2A), which focuses on the vascularization of lesions. The HFUS‐BW architecture uses a similar network to the aforementioned but with images from Dataset 2 (Figure 2B), capturing the morphology of the lesions.

**Figure 3:**
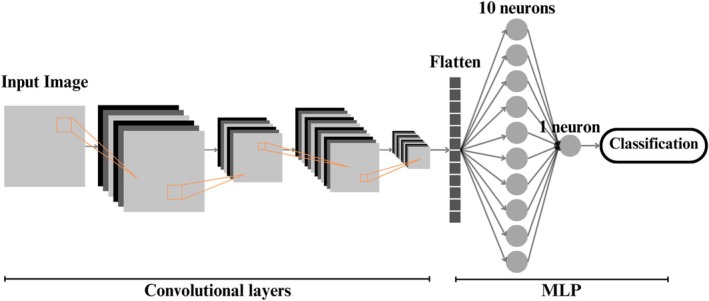
The basic architecture is used in the following networks: HFUS‐Doppler, HFUS‐BW, and Unity. The input image passes through two sequential convolutional layers. This is then flattened and connected to an MLP network consisting of a hidden layer and an output layer responsible for binary classification.

The architectures comprise two convolutional layers with progressive filter sizes; both use the ReLU activation function and are followed by pooling layers (MaxPooling) to reduce dimensionality. Following the convolutional operations, the data is flattened in an intermediate dense layer comprising 10 neurons, before being sent to the output layer comprising a single neuron with sigmoid activation. This layer produces the probability of the target class, as illustrated in Figure 3.

The network was trained using the binary loss function (*binary_crossentropy*) and an Adam optimizer with a learning rate configured for dynamic exponential decay adjustment. To address the imbalanced classes of the dataset, weights were assigned to each class based on their observed frequency during training. Training was carried out over 100 epochs with a batch size of 32 samples. The model's performance was monitored using the validation data, and the hyperparameters were adjusted accordingly.

#### Unity Architecture

The Unity architecture was designed to combine information from images acquired in both modes into a single input for the model. This approach simultaneously captures the morphological and vascular characteristics of lesions, enabling the model to learn from both imaging modalities together using Dataset 3.

The Unity model's network architecture is similar to those used for the separate B‐mode and Doppler mode networks, as shown in Figure 3. It maintains two convolutional layers with ReLU activation, pooling layers for dimensionality reduction, and a final dense layer with sigmoid activation for binary classification. The main difference lies in the input data: in the case of Unity, the images are concatenated. The aim of this model is to assess whether explicitly fusing information from both modes improves performance compared with networks that use the modes individually.

#### Cascade Architecture

**Figure 4:**
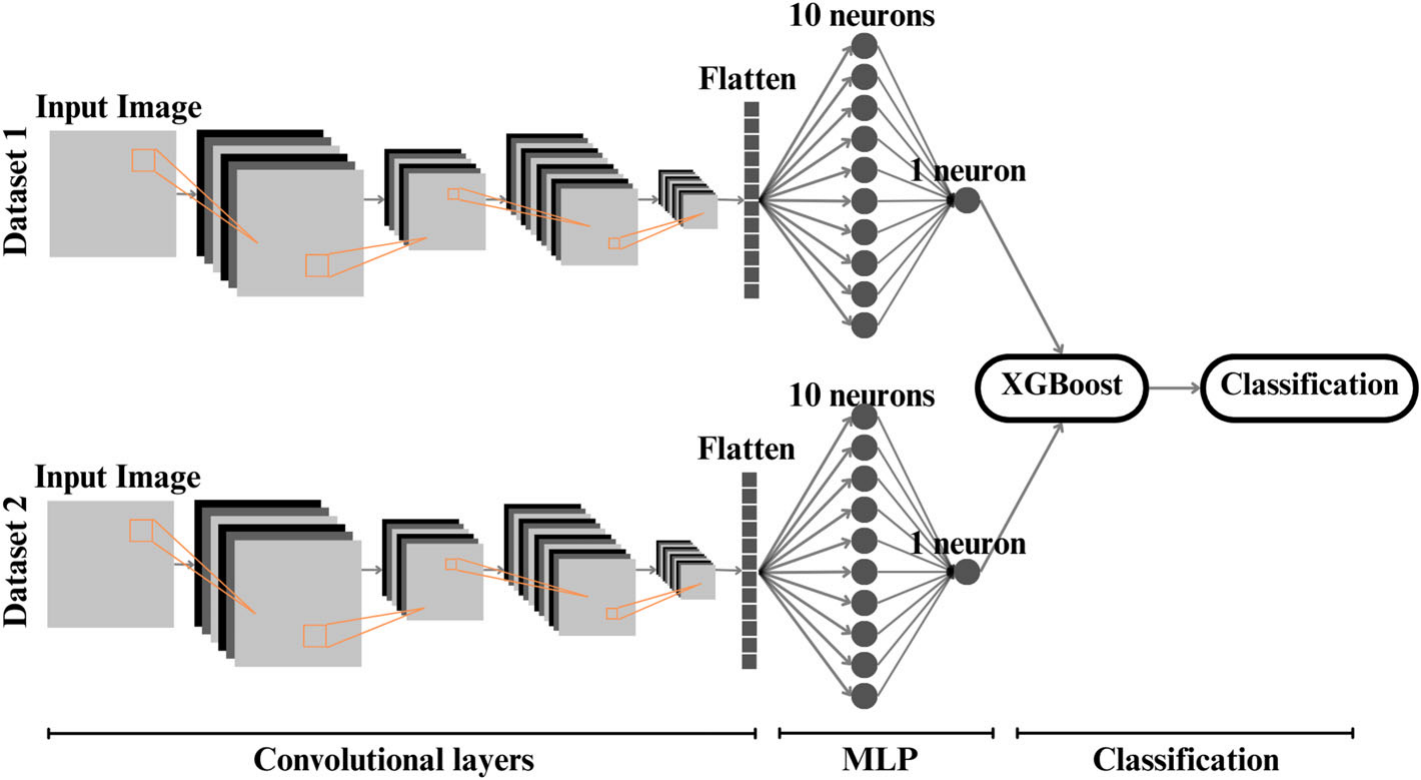
The Cascade architecture was used to classify skin lesions from datasets 1 and 2. Each input is processed individually by a convolutional network comprising two layers. These outputs are then flattened and processed by a MLP with a hidden layer, resulting in a single output neuron for each path. These two outputs are then concatenated and used as input for an XGBoost classifier, which makes the final decision.

The Cascade architecture combines two CNNs with two Multilayer Perceptron (MLP) layers and a classification model based on the Extreme Gradient Boosting (XGBoost) algorithm. This provides a hierarchical approach to classifying dermatological lesions (see Figure 4). Each CNN follows the same architecture as the networks described above for B and Doppler modes, with one trained exclusively on B‐mode images and the other on Doppler mode images. After training, the probabilistic outputs of these networks are extracted and used as inputs for the XGBoost classifier. These outputs represent the confidence of each model in the binary classification.

XGBoost is a machine learning algorithm based on decision trees that uses an ensemble technique to improve model performance. It is renowned for its efficiency and accuracy, and is widely used in classification and regression tasks. XGBoost optimizes the loss function using the gradient boosting method and incorporates regularization to avoid overfitting.

### Evaluating Metrics

The following metrics, calculated on the test set, were used to assess the performance of the proposed architectures: accuracy, recall, specificity, F1‐score, the receiver operating characteristic (ROC) curve, and the area under the ROC curve (AUC). These metrics are widely used in classification tasks, particularly in healthcare where clinical decisions depend on the reliability of predictive models. Together, the metrics provide a comprehensive assessment of the model's performance, taking into account both the detection of critical cases and the reduction of false diagnoses. In the healthcare context, an effective model must balance these aspects to provide reliable support for clinical decision‐making.

In addition, the *k*‐fold cross‐validation technique was used with k=5 to validate the models. This approach enables a more comprehensive assessment of performance by exposing the model to different subsets of the data. This approach enhances generalization reliability by mitigating the bias introduced through arbitrary partitioning of data into fixed training and test sets.

## Results

### HFUS‐Doppler and HFUS‐BW Networks

**Figure 5:**
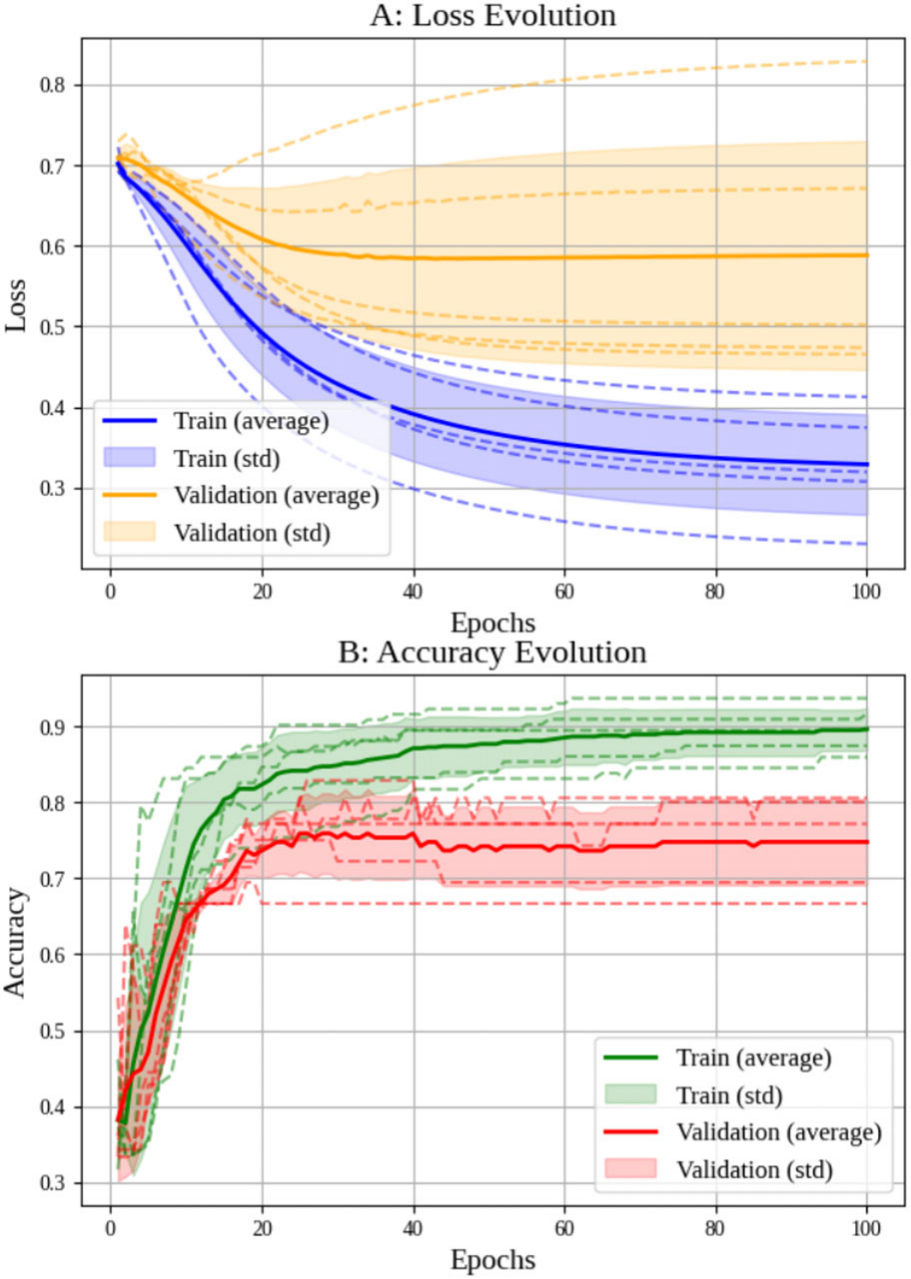
Results of *k*‐fold cross‐validation (*k* = 5) for the HFUS‐Doppler network. The upper graph shows how the loss function evolves over training epochs, while the lower graph shows how accuracy progresses.

**Figure 6:**
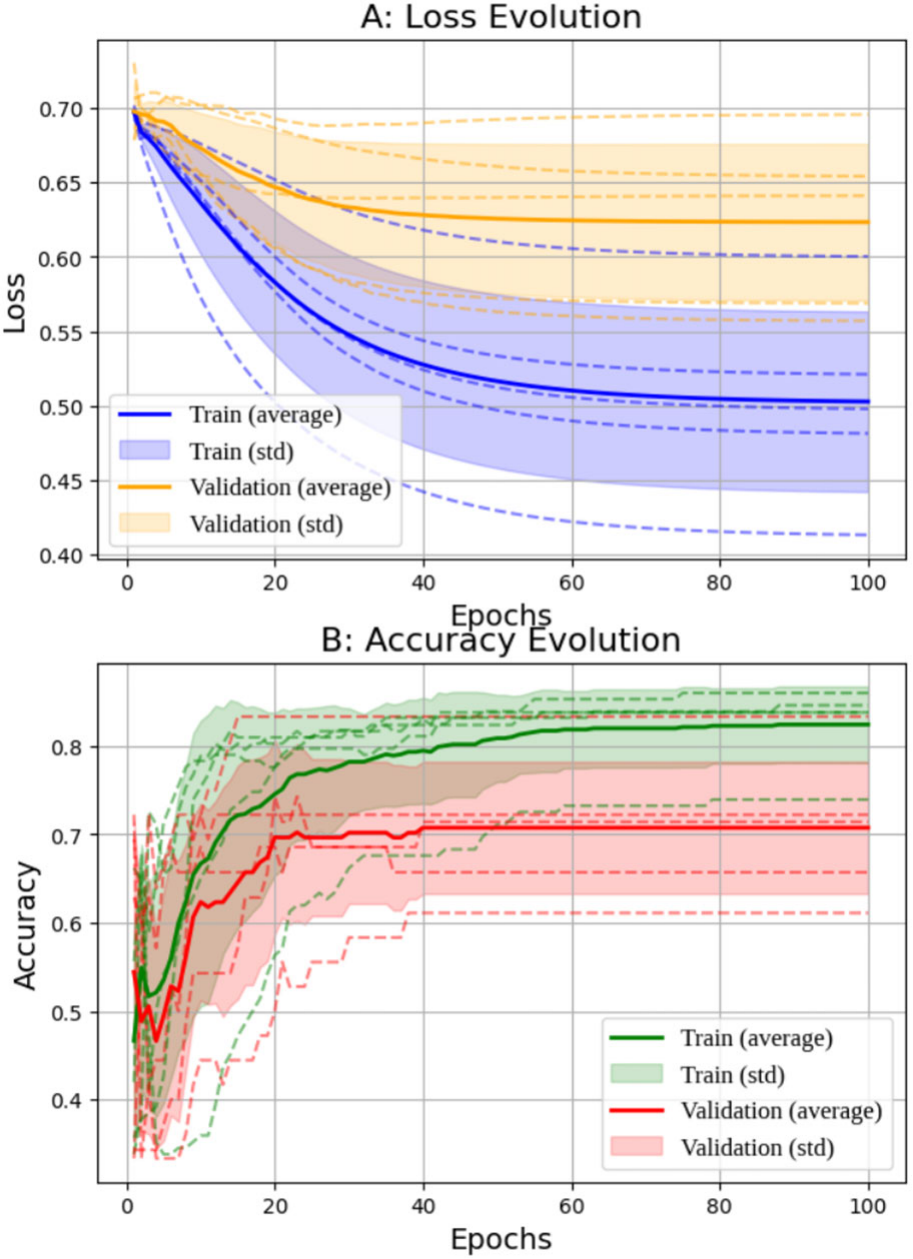
Results of *k*‐fold cross‐validation (*k* = 5) for the HFUS‐BW network.

Cross‐validation was performed on both networks (Figures 5 and 6), illustrating the loss and accuracy curves learned by the HFUS‐Doppler and HFUS‐BW models. The results show a consistent reduction in loss and an increase in accuracy over the training epochs.

**Figure 7:**
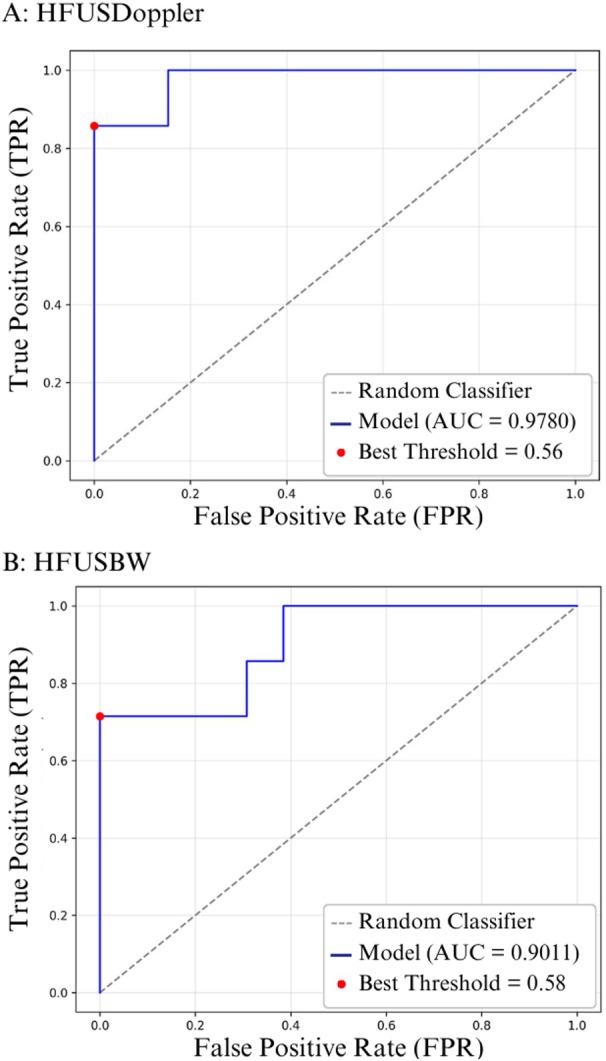
ROC curves of the evaluated networks. **A**, ROC curve of the HFUS‐Doppler network, with an area under the curve (AUC) of 0.9780; **B**, ROC curve of the HFUS‐BW network, with an AUC of 0.9011. The diagonal dashed line represents the performance of a random classifier. The blue lines correspond to the ROC curves of the models. The red dots show the optimal thresholds: 0.56 for the HFUS‐Doppler network and 0.58 for the HFUS‐BW network. These were determined based on Youden's *J* statistic.

Figure 7 shows the ROC curves for classifying the test dataset for the HFUS‐Doppler (A) and HFUS‐BW (B) models, respectively. For the HFUS‐Doppler network, the area under the curve (AUC) was 0.98, and the optimal threshold, as determined by Youden's *J* statistic, was 0.56. The HFUS‐BW network had an AUC of 0.90, with a best threshold of 0.58.

Using Youden's index to define the optimal threshold, accuracy, sensitivity, and specificity were calculated on the test dataset, which contains images that were not used during the training step. This evaluation highlights the models' ability to generalize to new data. The HFUS‐Doppler and HFUS‐BW networks achieved accuracies of 95% and 90%, respectively. A detailed breakdown of the remaining metrics is provided in the Table 1.

**Table 1:** The Evaluation Metrics Summarize the Generalization Performance of Each Network, as Measured on the Test Dataset

Metrics	HFUS‐Doppler	HFUS‐BW	Unity	Cascade
Accuracy	95.0%	90.0%	90.5%	85.0%
Recall	85.7%	71.4%	100%	100%
Specificity	100%	100%	83.3%	76.9%
F1‐score	92.3%	83.3%	90.0%	82.4%
AUC	0.98	0.90	0.97	0.92

**Figure 8:**
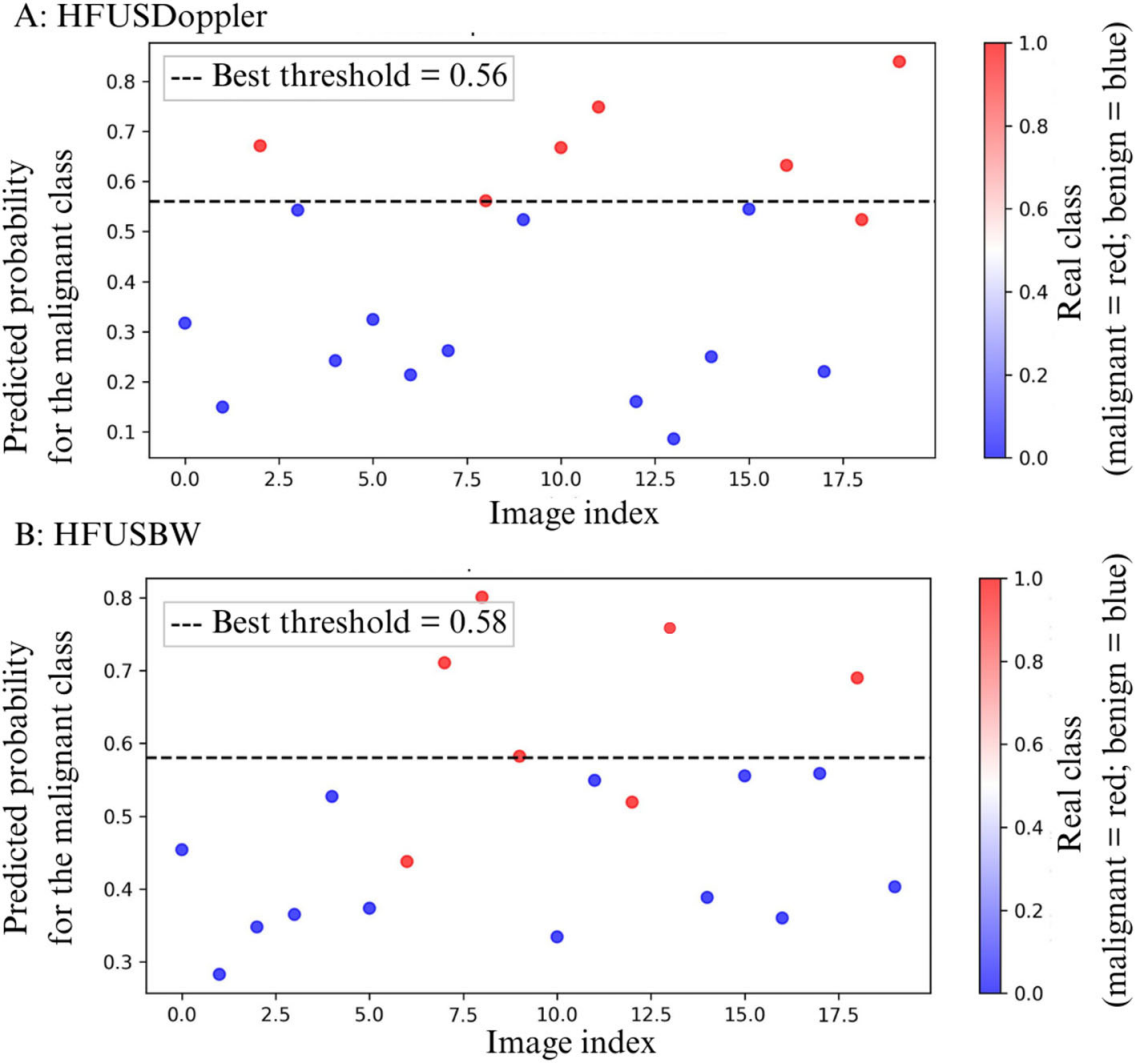
These are the classification results obtained using the test dataset with the HFUS‐Doppler (**A**) and HFUS‐BW (**B**) networks. The vertical axis shows the predicted probability of malignancy for each test image (indexed on the horizontal axis). The dashed line indicates the optimal decision threshold. Each point represents a sample from the test set, with the true class indicated by the color: red for malignant and blue for benign.

Figure 8 shows the performance of the networks in classifying the test data. Each point on the graphs represents the actual classification of a test set, and the black dashed lines represent the optimal thresholds determined by Youden's *J* statistic for classification. Points above the thresholds represent classifications as malignant, whereas points below represent classifications as benign. The color of the dots indicates the actual classification: red indicates a malignant lesion and blue indicates a benign lesion.

### Unity Network

**Figure 9:**
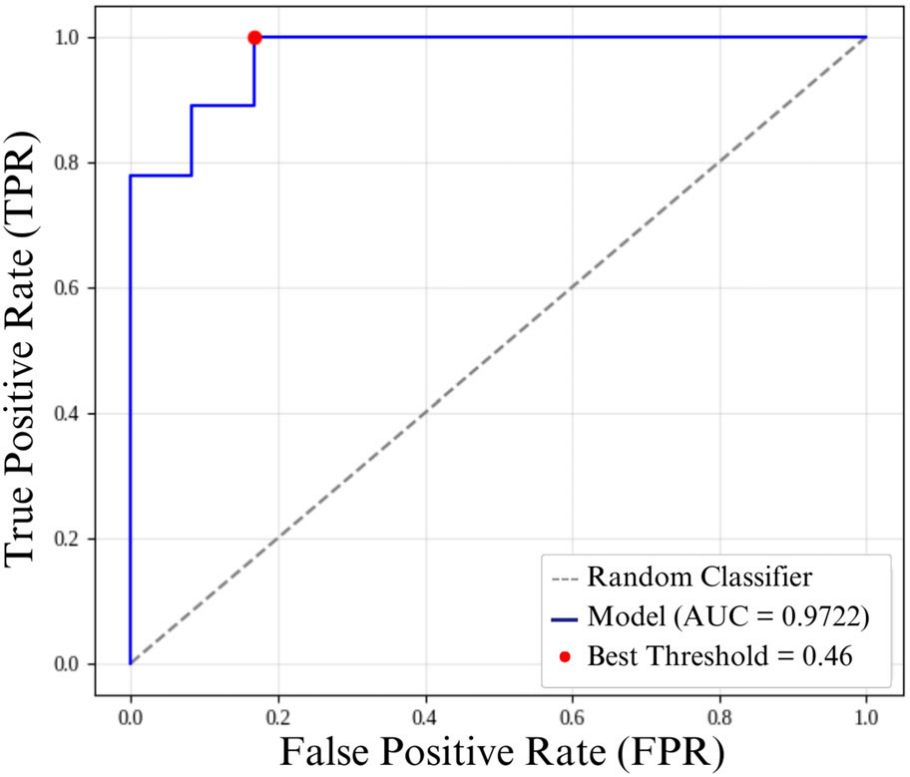
ROC curve for the Unity model using the test dataset. The red dot indicates the optimal threshold determined by Youden's *J* statistic.

**Figure 10:**
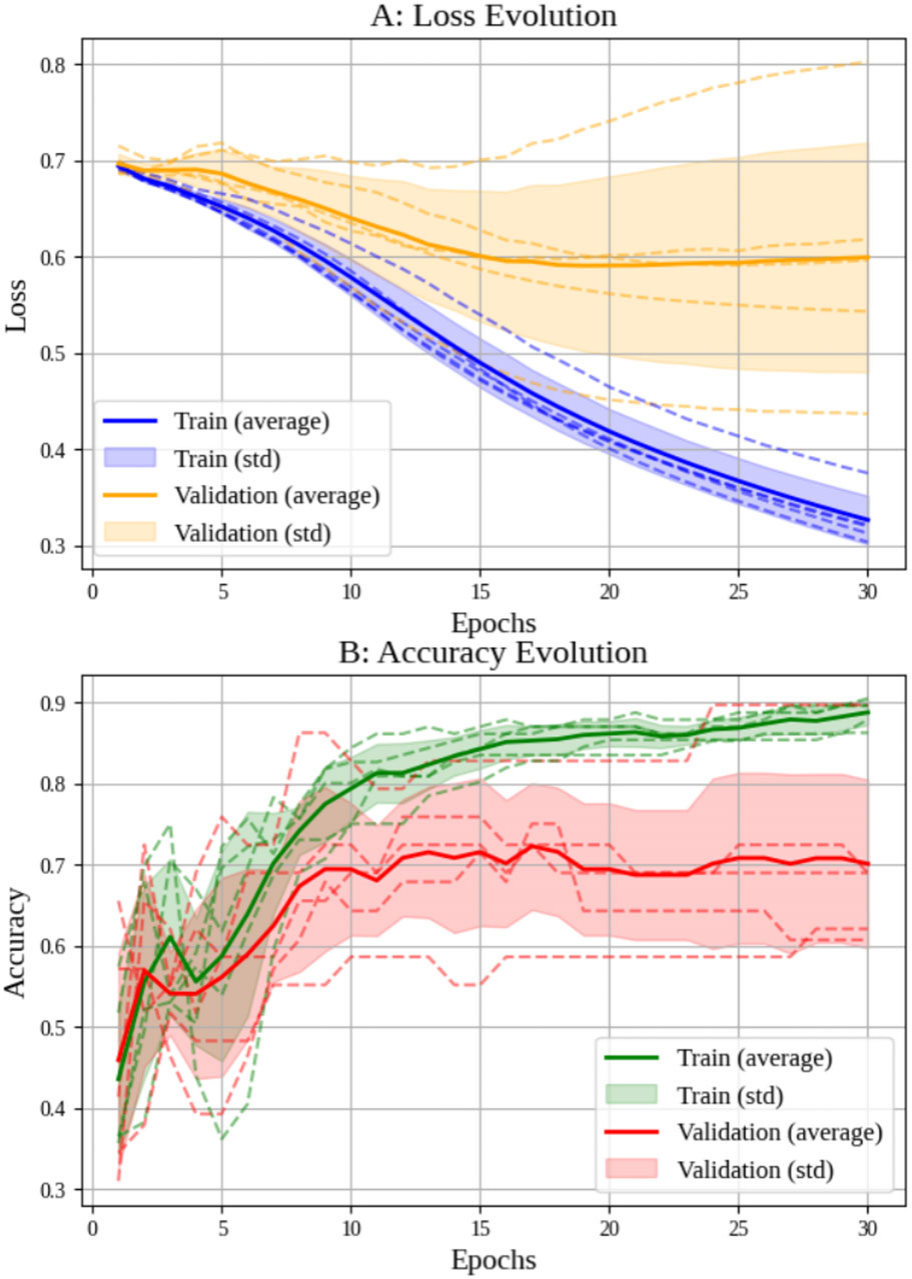
These are the results of *k*‐fold cross‐validation (*k* = 5) for the Unity model.

The Unity architecture, which combines B‐mode and Doppler images, outperformed the individual architectures. This model achieved an accuracy of 90.5% and an AUC of 0.97, as seen in Figure 9. The learning curves demonstrated convergence between the training and validation sets, with the validation curve occasionally exceeding the training curve without indicating overfitting (Figure 10).

### Cascade Network

**Figure 11:**
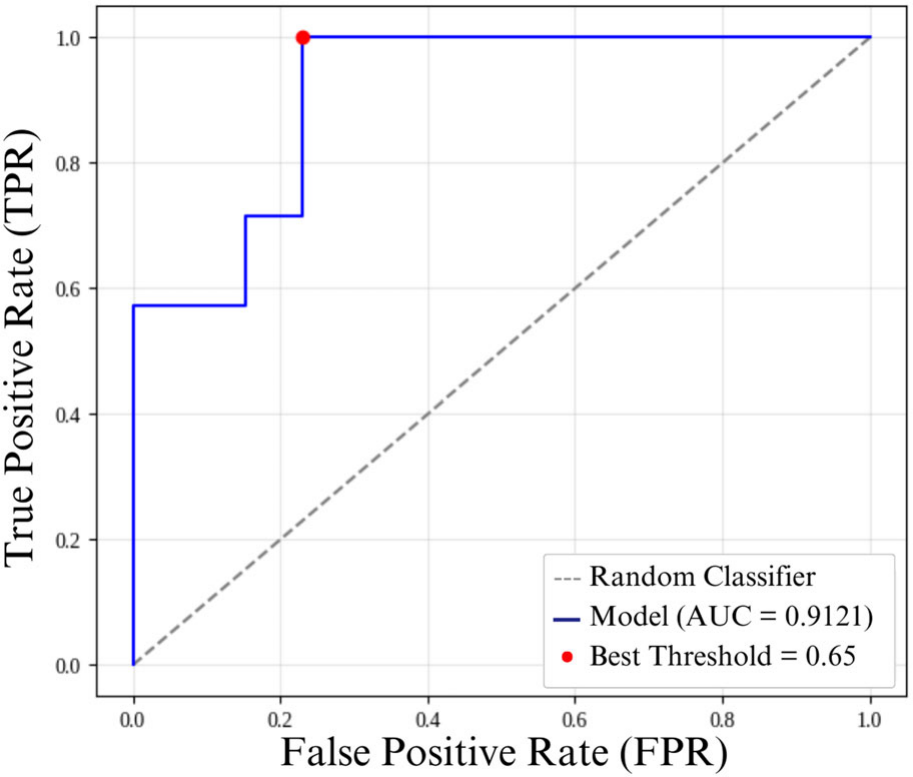
ROC curve of the Cascade model on the test dataset, with the optimal threshold defined by Youden's *J* statistic.

**Figure 12:**
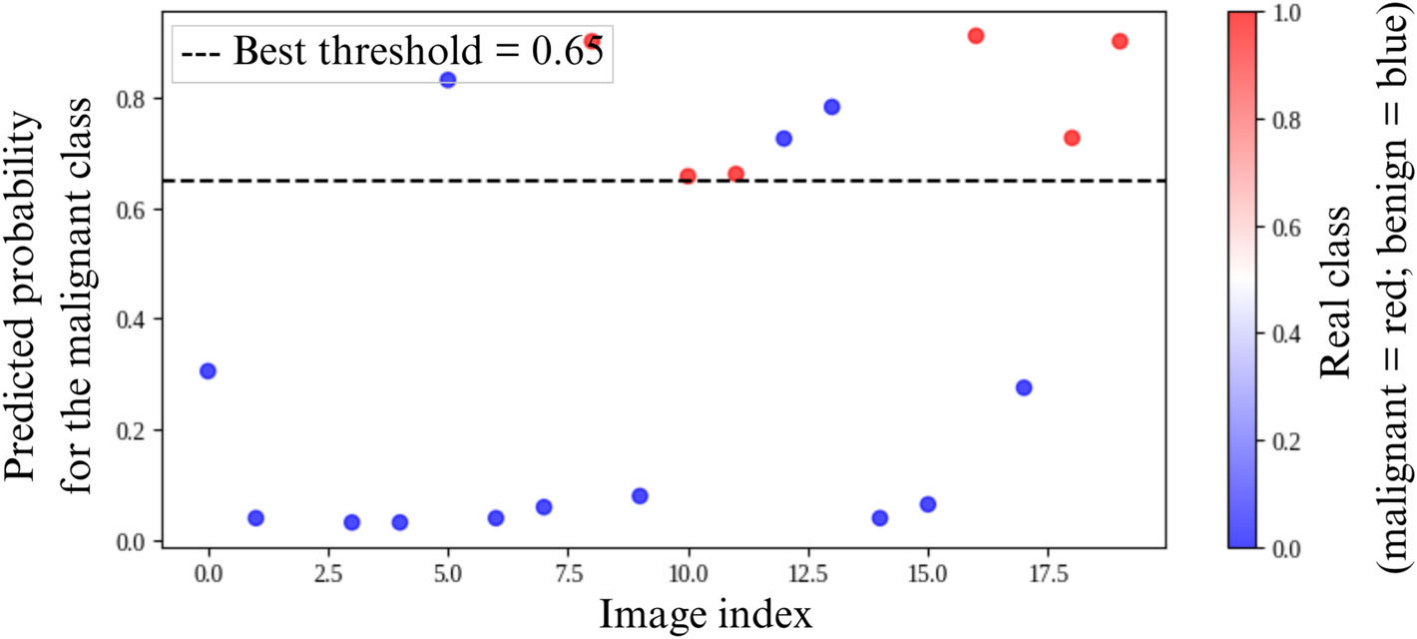
Classification of the test dataset samples by the Cascade model, with the optimal threshold indicated by Youden's *J* statistic.

As shown in Figure 4, the Cascade architecture, comprising two convolutional neural networks followed by an XGBoost classifier, achieved an accuracy of 85%, a specificity of 76.9%, and an AUC of 0.92 (Table 1). Due to the sequential nature of the Cascade design, cross‐validation was not applicable. Therefore, this model is evaluated independently from the others. The ROC curve presented in a, Figure 11 includes the optimal threshold of 0.65, as determined using Youden's *J* statistic. As depicted in Figure 12, the model exhibited a broader separation in the distribution of output probabilities, indicating higher discriminative capacity. Most predictions were placed farther from the decision threshold, suggesting that the model produced more confident classifications when compared with the other evaluated architectures.

## Discussion

As reported in the study by Laverde‐Saad et al,^4^ which used the associated dataset,^9^ the EfficientNet B4 network performed worse than models developed for the same classification task using the same dataset. Specifically, the network developed by Laverde‐Saad et al^4^ achieved an accuracy of 77.1%, a specificity of 80%, and a sensitivity of 73.3%. By contrast, the least accurate models in our study achieved accuracies of 85%. Furthermore, unlike the methodology adopted in the aforementioned study, all networks in this work, except for the Cascade model, were evaluated using cross‐validation to ensure adequate training and generalization.

AI has been widely explored in the medical imaging field for diagnostic support. However, in the specific context of dermatologic ultrasound, only a limited number of studies have investigated multimodal deep learning architectures.^7^, ^8^, ^11^ Most of these works rely on private datasets, which restrict reproducibility, whereas our approach employs a publicly available dataset, enabling transparency and replication of results. Furthermore, previous multimodal approaches have primarily combined clinical and B‐mode ultrasound images for lesion classification,^8^, ^11^ without incorporating Doppler information. Doppler imaging, however, provides essential vascular and hemodynamic data that is fundamental for comprehensive skin lesion evaluation, as emphasized in established dermatologic ultrasound guidelines such as the DERMUS consensus.^10^, ^12^ In this regard, our study reinforces the importance of integrating Doppler data into deep learning frameworks, demonstrating that multimodal models combining B‐mode and Doppler inputs achieve performance comparable to the current state of the art, while maintaining the advantage of being developed and validated on a reproducible, publicly accessible dataset.

The metrics presented in Table 1 indicate that the HFUS‐Doppler network demonstrated excellent generalization performance in the classification of skin lesions. With the highest accuracy (95.0%) and the highest area under the ROC curve (AUC = 0.98) among all evaluated models, HFUS‐Doppler stood out as the most effective approach. The Unity model also achieved strong results, with 90.0% accuracy, a high specificity and a high F1‐score, highlighting its robustness compared with the EfficientNet B4 model. In contrast, the Cascade architecture, despite incorporating alternative strategies, yielded lower performance. These findings underscore the importance of optimizing network architectures and fusion strategies to achieve an appropriate balance between recall and specificity, which is critical in medical imaging applications where false negatives may lead to delayed or missed diagnoses.

**Figure 13:**
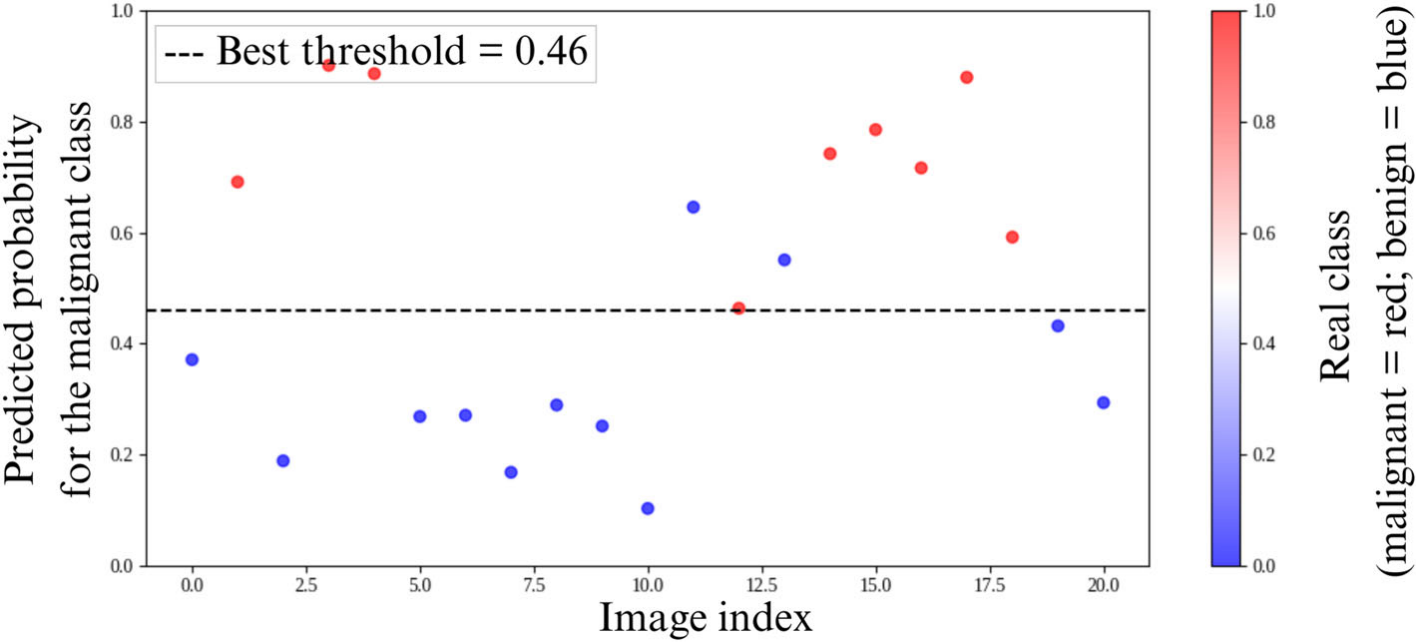
Classification of the test dataset samples by the Unity model, with the optimal threshold indicated by Youden's *J* statistic.

Figures 8, 12, and 13 show the predicted probability distributions for the HFUS‐Doppler, HFUS‐BW, Cascade, and Unity models, respectively, using the optimal thresholds identified via Youden's *J* statistic. The Cascade model, while performing worse in terms of metrics, generated probabilities that were closer to the extremes, indicating more assertive (albeit less accurate) classifications.

Analysis of the probability distribution graphs (Figures 8, 12, and 13) shows that the performance of the model should not be interpreted based solely on a fixed decision threshold. A better understanding can be gained by considering threshold ranges. While each network has an optimal cut‐off point, as determined by Youden's *J* statistic (dashed line), several samples are concentrated in intermediate probability regions close to the threshold. This characterizes a zone of uncertainty.

This zone of uncertainty is particularly relevant as it indicates situations in which the model is less confident in its predictions. Therefore, rather than adopting a single decision point, a strategy based on threshold ranges can be employed, whereby samples positioned in the high‐confidence zone (extreme probabilities close to 0 or 1) can be automatically classified with greater certainty. Conversely, samples located in the uncertainty zone (probabilities close to the optimal threshold) should be forwarded for further review by another model or human expert. This approach is particularly important in medical applications, such as diagnosing skin cancer, where false negatives or positives can have serious clinical consequences. Therefore, analyzing probability graphs confirms the networks' good overall performance and highlights the importance of incorporating uncertainty detection mechanisms into the decision‐making process to increase the robustness and clinical safety of predictions.

The results obtained by the different proposed architectures provide valuable insights into their potential for classifying dermatologic lesions in ultrasound images. Unity and Cascade models represent promising approaches that integrate morphological and vascular features extracted from B‐mode and Doppler acquisitions. However, the quality and consistency of the dataset remain an important factor for performance.

Despite forming the basis for model development and evaluation, the dataset presented limitations, including metadata inconsistencies and errors in image labeling. Some B‐mode and Doppler images were mislabeled or duplicated, and other records contained incorrect data. These irregularities required manual correction and led to the exclusion of samples, reducing the final dataset size. These limitations directly impacted model performance. For networks, especially those relying on fusion, to reach their full potential, a more robust image dataset is needed, with greater volume, higher quality, and better class balance. Ensuring equal representation of benign and malignant lesions is critical for effective learning and generalization. Future studies should focus on assembling larger, high‐quality datasets with rigorous quality control to minimize variability and support the development of reliable AI models for clinical applications.

Additionally, the dataset included lesions with markedly different characteristics, such as cysts, lipomas, and skin tumors, which may limit the model's ability to generalize across more specific diagnostic tasks. Future studies should therefore consider including more homogeneous and overlapping lesion types, particularly different kinds of skin tumors, to better assess the model's diagnostic accuracy in distinguishing benign from malignant lesions, such as nevi and melanoma.

In summary, the experimental results highlight the advantages of combining B‐mode and Doppler information for the classification of skin lesions using ultrasound images. Among the evaluated models, HFUS‐Doppler and Unity demonstrated the most robust and reliable performance. However, the findings also reveal that more complex fusion architectures do not necessarily lead to better results, particularly when trained on datasets with inconsistencies. Future research should focus on refining methods for combining complementary imaging data and expanding high‐quality and accurate datasets, which are essential for enhancing the reliability of clinical applicability of AI‐assisted dermatologic ultrasound diagnostics.

## Data Availability

The data that support the findings of this study are available in Dermatologic Ultrasound Images for classification at https://www.kaggle.com/datasets/alfageme/dermatologic‐ultrasound‐images?select=201database.csv. These data were derived from the following resources available in the public domain: Kaggle, https://www.kaggle.com/datasets/alfageme/dermatologic‐ultrasound‐images?select=201database.csv.
